# Weight, gender, and depressive symptoms in South Korea

**DOI:** 10.1002/ajhb.22972

**Published:** 2017-02-05

**Authors:** Alexandra A. Brewis, Seung Yong Han, Cindi L. SturtzSreetharan

**Affiliations:** ^1^ School of Human Evolution and Social Change Arizona State University Tempe Arizona 85284‐2402; ^2^ Obesity Solutions Arizona State University, Arizona State University Tempe Arizona 85284‐2402

## Abstract

**Objectives:**

Obesity consistently predicts depression risk, but the underlying mechanisms are poorly understood. Body concerns are proposed as key. South Korean society is characterized by extremely high levels of explicit weight stigma, possibly the highest globally. Using cross‐sectional Korean 2014 National Health Examination Survey (KNHANES) data, we test this proposition in a nationally representative sample of South Korean adults (*N* = 5,632).

**Methods:**

Depressive symptoms (outcome variable), was based on the PHQ‐9. Weight status (predictor variable), was based on direct measures of height and weight converted to BMI. Weight concern was self‐reported. Mediation analyses tested how weight concern mediated the influence of weight status on depressive symptoms for women and men.

**Results:**

Current weight status influenced depressive symptoms in Korean adults, but not always directly. Concerns of being “fat” mediated that relationship. The effect increased significantly as BMI increased within “normal” and overweight/obese categories for women, and in overweight/obese categories for men. Even though women classified as underweight were significantly more depressed than those in other weight categories, there was no similar mediation effect related to weight concerns.

**Conclusion:**

For South Koreans, the stress of adhering to social norms and avoiding stigma related to body weight seems to explain the relationship between higher body weight and more depressive symptoms. Women are more vulnerable overall, but men are not immune. This study demonstrates that body concerns help explain why weight predicts depression, and more broadly supports the proposition that widespread weight‐related stigma is a potentially major, if unrecognized, driver of population‐level health disparities.

## 1 Introduction

Social stigma is proposed as a major unrecognized driver of population‐level health disparities, one vastly understudied as a key aspect of human health and biology (Hatzenbuehler, Phelan, & Link, 2013). A growing set of literature is considering how feeling “too fat,” exacerbated under social conditions of high weight stigma, might result in an array of negative health outcomes like weight gain and depression—with women especially at risk (see Major, Tomiyama, & Hunger, 2017 for a recent review). This theorized relationship is based almost entirely on analysis of data drawn from the US, Western Europe, and Australia, especially undergraduate college student samples. With the rise of overweight/obesity as a common aspect of contemporary human biology, and the concurrent rise of anti‐obesity public health efforts, weight stigma is globalizing (e.g., Brewis, Wutich, Falletta‐Cowden, & Rodriguez‐Soto, 2011; Marini, et al., 2013). Yet, we do not know if weight stigma might also exert significant influence on stress‐related health outcomes outside of the Anglosphere (*cf*. Hackman, Maupin, & Brewis, 2016; Maupin and Brewis, 2014), and if the risk is similarly feminized.

Depressive symptoms provide a suitable lens to examine how complex, dynamic, stressful social exclusionary processes like weight stigma become embodied as worse health. First, at the population level depressive symptoms correlate with many everyday struggles and insecurities, such as poverty and chronic ill health (e.g., Moussavi, Chatterji, Verdes, Tandon, Patel, & Ustun, 2007). But, importantly to the analysis of stigma, depressive symptoms are highly sensitive to an array of social exclusions, including subjective and objective experiences of discrimination and social rejection and isolation (for example of recent review, see Schmitt, Branscombe, Postmes, & Garcia, 2014). Second, a substantial epidemiological and public health literature has identified a bidirectional relationship between obesity and depression that is especially evident among women. While studies to date are few, body/weight concern issues have been identified as an important potential mediator that may help explain the gendered pattern of risk; in fact, it is the only variable that consistently predicts both depression and obesity (see Preiss, Brennan, & Clarke, 2013 for a full review).

Moreover, biocultural studies have shown that depression symptoms are often gendered because women and men inhabit different social niches that expose them differentially to local‐level injustices and other social, economic, and political exclusionary processes (e.g., Hadley and Wutich, 2009; Hadley, Brewis, & Pike, 2009). Some prior reviews (Atlantis and Baker, 2008; Markowitz, Friedman, & Arent, 2008; Preiss, Brennan, & Clarke, 2013) suggest that gender only influences the relationship between obesity and depression/depressive symptoms in the U.S. specifically because of the “special” high value placed on body perfection. Certainly, U.S. women are at significantly greater risk of feeling weight‐related stigma and for body concern‐related depression than men, in part because the social norms of slimness are so tied to femininity and greater female body capital/upward mobility (Anderson‐Fye and Brewis, 2017; Brewis, 2014).

If weight stigma matters to a more globalized human biology as a consistent population‐level driver of health disparities, its depressive effect should manifest strongly in any setting where weight stigma is highly evident. Our study relating weight and weight concern to depression is focused on South Korea due to its recent identification as possibly the most explicitly weight‐stigmatizing of all nations (Marini, Sriram, Schnabel, Maliszewski, Devos, Ekehammar, … Schnall, 2013). According to the OECD report on obesity (2014), while obesity rates remain comparatively very low in South Korea (2% in 1995 and 4% obese in 2011), overweight is on the increase overall (23% in 1995 and 28% overweight in 2011). In addition, an increase in obesity rates among children and adolescents are raising concerns in recent years, especially the accelerating rates among male students (Lim, Park, & Gu, 2009).

In South Korea, powerful social norms emphasize the need for maintaining thinness as a key to self‐presentation (Han, 2003; Kim, 2014). Indeed, South Koreans are considered to be preoccupied with dieting and body size, regardless of their own actual weight (Han, 2003). Comments emphasizing weight are common in everyday discourse (Kim, 2014; Schwekendiek, Yeo, & Ulijaszek, 2013). Korean media makes large bodies a focus of degrading humor (Lim and Kim, 2012), and images are replete with tall, muscular men and thin, beautiful women (Kim, 2005). Pike, Hoekb, & Dunnee (2014) have suggested that the level of disturbed eating and body dissatisfaction in South Korea is one of the highest in the world. Prior studies have identified body issues as a trigger for other forms of psychosocial distress in South Korean adolescents specifically, such as suicidal ideation (Kim, 2009; Kim and Kim, 2009; Kim, Cho, Cho, & Lim, 2009), eating disorders (Choi, Choi, Park, Joo, Ga, Ko, & Kim, 2007), and depression (You et al., 2016).

It is also notable that the ideals of the slim South Korean body is not one simply borrowed from the West but is shaped by its own traditionalist and nationalist concerns (Holliday and Elfving‐Hwang, 2012). Cosmetic surgery is a socially acceptable means to achieve one's desired body image. South Korea has the highest rates globally, and at times surgery has been tax deductible. This could quite differently shape the relationship between a highly weight‐stigmatizing social context and its embodied consequences (as represented by depressive symptomatology). For example, stringent beauty ideals combined with easy access to cosmetic surgery contribute to highly inflexible body norms among contemporary South Korean women and men. This may amplify greatly the depressive effects of body concerns because the norms are so narrowly defined, making it harder for people to approximate them.

The gendered pattern of risk, with women predominating, might be less evident in South Korea, demonstrating that men are susceptible to the social processes in ways not previously observed elsewhere. Korean men are reportedly highly concerned with their looks (Schwekendiek et al., 2013). By comparison with the West, male cosmetic surgeries are on the rise. They are not seen as fundamentally feminizing nor considered a threat to masculinity (Holliday and Elfving‐Hwang, 2012; Swami, Hwang, & Jung, 2012). This fits with a broad distinction between cultural notions of masculinity between Northeast Asian cultures and Western cultural notions; in Japan, for example, body modification and body work by men has been considered not only appropriate but practically common sense for the past decade (Miller, 2006). Representations of ideal masculinity related to very specific body presentations abound in social media. These masculine ideals are actively pursued and obtained by everyday people (Hiramoto, 2015; SturtzSreetharan, in press). While Joh, Oh, Lee, & Kawachi, (2013) found that South Korean men are more likely to under‐estimate their weight than women, both male and female students, elementary through university, are found to exhibit the types of distorted body image associated with disordered eating (Hong, Jung, Kim, Lee, Hyun, Bahk, … Lee, 2015; Jung & Forbes, 2006). Among South Korean adolescents, overweight males have been reported as more likely verbally to bully and be bullied than their female counterparts (Kim, Yun, & Kim, 2016).

Our goals for the analysis reported here were to (a) identify how body size (BMI) predicts level of depressive symptoms in the general adult population of South Korea, (b) test if concerns about being “fat” are a crucial mediator of that relationship, and (c) determine if Korean men might be similarly vulnerable to the depressive effects of such weight concern as women.

Beyond this, one recent study from Guatemala (Hackman et al., 2016) has shown that (unlike what is typically observed within the U.S. and similar contexts), weight stigma‐related depression can be observed at both ends of the weight spectrum, including among those of lower body weights who are concerned about being too thin. This suggests cross‐cultural analyses assessing the health impacts of weight stigma should also consider if body concerns about being too thin might be similarly stressful on mental health. We thus also (d) test if similar mediation effects are evident around concerns of being “thin” among those classified as such by their body weights.

## 2 Methods

### 2.1 Data

Our study employed the Korean 2014 National Health Examination Survey (KNHANES) data to test the relationship between weight status, body concerns, and risk for depressive symptoms in a nationally representative sample of South Korean adults (KCDC, 2014; Kweon et al., 2014). The Korean National Health Examination Survey (KNHANES) was first collected in 1998 and designed to examine the health and nutrition status of children, adolescents, and adults living in South Korea in different time periods until 2015. It was based directly on the US‐NHANES. Out of the total 7,550 cases in 2014, only those respondents who were ≥19 years old (the KNHANES definition of “adult”) were included in the sample. The final sample size was 5,632 after removing cases with missing values in the variables used for analysis. The 2014 dataset and study materials are currently publically available for download in Korean Hangul.

### 2.2 Measurement

Weight status, the predictor, was classified based on directly measured weight and height, converted to Body Mass Index (BMI). Height was measured with a portable stadiometer, and weight with a Seca electronic scale in normal clothing. BMI < 18.5 was classified as underweight, 18.5 ≤ BMI < 25 as “normal,” 25 ≤ BMI < 30 as overweight, and ≥30 as obese, per convention. Given the low numbers of South Korean adults categorized as obese in the sample (BMI ≥ 30, 4% for both genders, matching national estimates), we collapsed overweight/obese into a single category (BMI ≥ 25 as overweight/obese). This collapsed variable is further justified by the observation that BMI measures tend to underestimate adipose levels in Asian populations compared to those in other regions (Hruschka and Hadley, 2016).

The level of depressive symptoms (the outcome variable) was measured using the 9‐item Patient Health Questionnaire (PHQ‐9) that asks survey respondents to consider the two weeks prior to the survey when selecting an answer (Kroenke, Spitzer, & Williams, 2001). The PHQ‐9 is a multipurpose instrument for screening, diagnosing, monitoring, and measuring the severity of depressive symptoms, with possible scores ranging from 0 to 27. The PHQ‐9 Korean version has been validated previously with several populations, including older adults (Han, Jo, Kwak, Pae, Steffens, Jo, & Park, 2008), clinical patients (Seo and Park, 2015), students (Yoon, Lee, Pae, Yoon, Patkar, Steffens, & Kim, 2014) and Korean‐Americans (Shin, Park, Cho, Chiu, Bang, H., & Bernstein, 2010); a score of 10 or higher has been validated in clinical studies as defining depression (Kroenke et al., 2001). Since this scale was included only in the 2014 wave, we could not include prior KNHANES data sets in the analysis.

Weight concern, the hypothesized mediator, was classified based on people's self‐reports (i.e., they said they were very thin (매우 마른 편), a bit thin (약간 마른 편), normal (보통), a bit obese (약간 비만), or very obese (매우 비만)). The variable has a range between zero and two, with zero point if “very thin”, “a bit thin”, or “normal”, one point if “a bit obese”, and two points if “very obese.” For modeling those in the underweight category, the directionality was reversed, so zero points were given if a respondent perceived their own weight as “very fat”, “a bit fat”, or “normal”, one point if “a bit thin”, and two points if “very thin.”

The mediation model included a number of covariates known to influence depression symptoms. Health‐related factors included self‐assessed general health status (a continuous variable measuring how a respondent assesses his or her general health with 0 points as the worst and 4 points as the best), “stress” (a dichotomous variable measuring whether or not a respondent reported being “very stressed” in his or her regular daily life), and “chronic disease” (a dichotomous variable measuring whether or not a respondent reported managing high blood pressure, diabetes, or high cholesterol). A validated index for life quality (EQ‐5D) was included in the KNHANES survey with five items asking about the level of physical activity, health self‐management, ease of daily activity, pain or discomfort, and anxiety or depression. Since the level of depressive symptoms is the main outcome in this paper, the last item was excluded, and the other four sub‐items were included independently in the model. Each sub‐item has a range between zero and two with higher values indicating higher quality of life. Socioeconomic status (SES) was estimated on the basis of average monthly household income (Korean won), whether or not a respondent owned a house or an apartment, occupation status, the education level of a respondent, and the education level of each parent. Demographic factors included marital status and age. We also included weight change efforts in the previous year (attempting to gain, lose, or maintain weight, or doing nothing).

### 2.3 Analytic strategy

**Figure 1:**
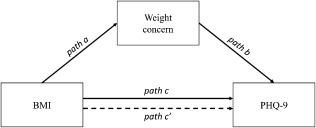
Mediation Model with Weight Concern as the Mediator, Korean Adults, 2014

Descriptive statistics (Table 1) were estimated using Procedure Survey mean in SAS 9.4 to take the survey design into account. To test if concern with being thin or being overweight or obese mediates the associations between BMI and the level of depressive symptoms, a statistical analysis with a single mediator model using multivariate delta method standard error was conducted by using Proc Survey reg in SAS 9.4 (MacKinnon, 2008). Analyses were weighted to represent the national population of South Korea (https://knhanes.cdc.go.kr/knhanes/eng/). The mediation model is visually summarized in Figure 1. In detail, the effect of BMI on weight concern (path a) and the effect of weight concern on the level of depressive symptoms (path b) were estimated by using ordinary least square regression models. Then, whether or not the product of two estimated coefficients (path a × path b) is significantly different from zero was tested at the *p*‐value .05 level. In the mediation analysis, BMI and weight concern were centered around their own means within each weight category (normal vs. overweight/obese). For better interpretability, mediation analyses were conducted within weight categories (i.e., for those classified by BMI as “normal” weight or overweight/obese. In the case of those classified as underweight (BMI < 18.5, *N* = 248), we did a separate analysis that reversed the weight concern gradient, testing if lower (rather than higher) BMI increments had a similar depressive effect, that is, considering concerns about being “thin” instead of “fat” as positive effects. Separate mediation models were run for women and men. Additional statistical analyses beyond consideration of relationships among key variables are provided in Supporting Information Tables S1–S6.

**Table 1:** Summary statistics of main variables by weight category, Korea adults, 2014

	Underweight		Normal weight		Overweight/Obese	
	Female	Male	Female	Male	Female	Male
Depressive Symptoms (PHQ‐9)	4.81 (0.52)	3.77 (0.57)	3.28 (0.11)	2.15 (0.11)	3.29 (0.18)	2.22 (0.15)
BMI	17.59 (0.08)	17.50 (0.10)	21.88 (0.04)	22.44 (0.05)	27.71 (0.10)	27.58 (0.10)
Weight concerns^a^	1.12 (0.05)	1.64 (0.07)	0.36 (0.02)	0.15 (0.01)	1.17 (0.03)	1.02 (0.03)
Health‐related factors						
Self‐assessed health	2.03 (0.07)	2.06 (0.14)	2.12 (0.02)	2.31 (0.03)	1.98 (0.03)	2.19 (0.04)
Stress	37%	32%	27%	22%	25%	25%
Chronic disease	6%	16%	21%	21%	42%	30%
*Life quality measures*						
Physical activity score	1.85 (0.03)	1.90 (0.04)	1.88 (0.01)	1.91 (0.01)	1.74 (0.02)	1.92 (0.01)
Self‐management score	1.97 (0.01)	1.95 (0.03)	1.96 (0.00)	1.98 (0.00)	1.93 (0.01)	1.98 (0.01)
Normal activity score	1.94 (0.02)	1.85 (0.05)	1.92 (0.01)	1.95 (0.01)	1.86 (0.01)	1.93 (0.01)
Pain or discomfort score	1.76 (0.04)	1.80 (0.06)	1.73 (0.01)	1.82 (0.01)	1.65 (0.02)	1.82 (0.02)
Socioeconomic factors						
Household income (times 10,000 won)	404.42 (37.97)	339.86 (52.57)	394.22 (15.97)	444.44 (29.13)	315.06 (15.69)	395.37 (16.36)
Own a house or an apartment	64%	55%	66%	67%	63%	65%
*Occupation status*						
Regular	16%	6%	11%	25%	6%	23%
Nonregular	25%	32%	23%	24%	20%	19%
Self‐employed or else	7%	11%	14%	21%	17%	27%
Not working or unemployed	52%	51%	53%	30%	57%	30%
*Respondent's education*						
Less than high school	9%	29%	24%	21%	44%	17%
High school graduate	41%	53%	38%	40%	36%	41%
College or above	50%	18%	37%	39%	20%	41%
*Parents’ education*						
College or above, father	24%	10%	14%	15%	6%	12%
College or above, mother	11%	8%	6%	7%	3%	5%
Weight management						
Tried to increase	22%	49%	3%	11%	1%	1%
Tried to decrease	10%	0%	44%	22%	63%	59%
Tried to keep or do nothing	68%	50%	52%	68%	36%	40%
Demographic factors						
*Marital status*						
Currently married	40%	53%	67%	68%	70%	74%
Never married	48%	41%	18%	27%	9%	23%
Divorced, widowed, or separated	12%	5%	15%	6%	20%	4%
*Age group*						
Between 19 and 29	46%	37%	18%	21%	10%	17%
Between 30 and 39	24%	10%	19%	18%	13%	23%
Between 40 and 49	14%	11%	22%	21%	18%	23%
Between 50 and 59	8%	15%	19%	19%	23%	22%
Over 60	9%	26%	22%	21%	36%	16%
*N* b	173	75	2,179	1,430	900	875

Note: mean and standard error of the mean in parentheses; a percentage if dichotomous (1/0); adjusted for sample weight.

a Weight concern is reversed (0 = normal, fat, very fat/1 = thin/2 = very thin)

b The full number of cases (preweight) including those with missing values in one or more variables since NOMCAR option in Proc Surveymean statement is used

## 3 Results

### 3.1 Summary statistics

In normal and overweight/obese categories, weight concerns showed a high positive correlation with BMI (correlation coefficient = 0.66, *p* < .0001), noting that 28% of women and 12% of men incorrectly estimated their weight category as higher than it was based on BMI. Considering underweight respondents only, the correlation is negative (correlation coefficient = −0.26, *p* < .0001), meaning that weight concerns went up and weight went down (and noting that 12% of women and 6% men classified by their weight in this category did not identify themselves as being underweight).

#### 3.1.1 Women

**Figure 2:**
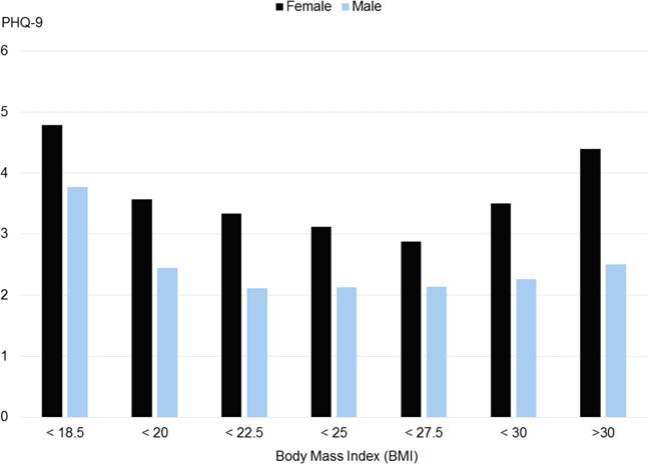
Level of Depressive Symptoms (PHQ‐9) by BMI, Korean Adults, 2014. Note: adjusted for sample weight; underweight (BMI < 18.5) is included in this figure to show the overall patterns; range of PHQ‐9 within each BMI category for female/male is 27/22, 27/15, 27/24, 27/22, 21/24, 27/18, and 22/14 (all start from zero) [Color figure can be viewed at wileyonlinelibrary.com]

In the total sample, 28% of women were categorized as overweight/obese (see Table 1 for summary statistics). The level of weight concern women expressed is significantly higher in the overweight/obese group compared with those categorized as normal weight (*p* < .0001), and a similar significant pattern related to weight concerns was observed in those classified as underweight related to being “too thin.” Depressive symptoms were in a U‐shaped relationship with body size, with higher values observed at both ends of a BMI distribution (see Figure 2). Compared with those in the normal weight category, those classified as underweight had significantly higher levels of depressive symptoms (*p* = .003). There was not a significant difference between those in overweight/obese versus normal weight categories (*p* = >.05).

#### 3.1.2 Men

Thirty‐seven percent of men were categorized as overweight/obese. As with women, weight concern was highest among those categorized as overweight/obese and underweight compared to those categorized as normal (*p* < .0001). However, between those categorized as underweight and overweight/obese, those categorized as underweight expressed slightly greater concern than the other category. The overall relationship of men's depressive symptoms as related to BMI was skewed toward underweight. For men, the level of depressive symptoms was significantly higher for those classified as underweight than normal (*p* = .005), and there was (as for the women) no significant difference in overall level of depressive symptoms between men classed as normal and those classed as overweight/obese (*p* = >.05).

### 3.2 Associations between covariates, weight concern, and depressive symptoms

The estimated effects of other modeled factors associated with weight concern and depressive symptoms are presented for each category in Supporting Information Tables S1–S6. Generally, results were as expected. For example, worse self‐assessed general health and greater reported everyday stress predicted more depressive symptoms regardless of gender and weight status. Less financial insecurity, lower household income, and other markers of lower SES also predicted greater risk of depressive symptoms for all weight groups except the underweight. Physically active respondents, those with fewer limitations to normal activity, and those experiencing less pain or discomfort tended to have lower levels of depressive symptoms, regardless of weight status.

### 3.3 Mediation analysis

#### 3.3.1 Women

Once covariates were taken into account, higher BMI measures directly predicted higher depressive symptoms (total effect *c*) for those women classified by their BMI as overweight/obese, but not for those classified as underweight or normal weight (see Table 2). However, this positive relationship between BMI and depressive symptom level in women is significantly mediated by degree of weight concern (*p* = .009 for the normal and *p* = .007 for the overweight/obese) for those classified as both normal and overweight/obese (mediated effect a × b). Women's greater weight concerns explain the observed correlation between higher BMI to more depressive symptoms within each weight status category.

**Table 2:** Mediation analysis results of the association between BMI (X) and PHQ‐9 (Y) with weight concern (M) as a mediator by weight status, Korea, 2014

		Underweight^a^		Normal weight		Overweight and Obese	
Effect	Path	Female	Male	Female	Male	Female	Male
Total (c)	X →Y	−0.275 (0.517)	−0.383 (0.701)	−0.077 (0.054)	0.068 (0.063)	**0.186 (0.057)**	0.014 (0.051)
Direct (c')	X (M)→Y	−0.139 (0.528)	−0.346 (0.729)	−**0.167 (0.062)**	0.097 (0.064)	0.117 (0.066)	−0.061 (0.063)
Path (a)	X→M	−**0.212 (0.061)**	−0.063 (0.080)	**0.166 (0.008)**	**0.075 (0.008)**	**0.092 (0.010)**	**0.120 (0.009)**
Path (b)	M→Y	0.639 (0.560)	0.590 (1.593)	**0.539 (0.204)**	−0.397 (0.221)	**0.748 (0.264)**	**0.617 (0.227)**
Mediated (a × b)	X→M→Y	−0.136 (0.125)	−0.037 (0.111)	**0.090 (0.034)**	−0.030 (0.017)	**0.069 (0.025)**	**0.074 (0.028)**

Note: **Bold** if the result is significant at least at the 0.05 level of significance; adjusted for sample weight; weight change, self‐assessed health, stress, household income, marital status, education, and age are controlled for; BMI and weight concern are centered around their own means within each weight group.

a Weight concern is reversed (0 = normal, fat, very fat/1 = thin/2 = very thin); adjusted for sample weight; See Appendix 1a, 1b, 2a, and 2b, for full results.

#### 3.3.2 Men

Among men, the same significant positive mediation effect of weight concern was also seen in those classified as overweight/obese, meaning that as BMI increased above 25 toward 30 so then did the effects of body concerns on depressive symptoms (*p* = .008). The mediated effect for men between BMI of 18.5 and 25 (“normal weight”) was negative, and so the reverse direction of that for women, and men had more depressive effects of body concerns as they approached underweight BMIs of <18.5 within this category. However, this was not statistically significant (*p* > .05). There was no significant mediation effect for men classified as underweight (*p* > .05; see Supporting Information Tables for further details).

## 4 Discussion

Alongside low national levels of obesity, South Korean beauty standards are stringent and normative ideals focus on the need for slim physical body presentation. Using a large nationally representative sample of adults, we found that body size predicts level of depressive symptoms. This analysis of nationally representative data from South Korea demonstrates that current weight status (BMI) influences depressive symptoms, but not necessarily directly. Specifically, concern about being “obese” (비만) is a significant mediator among both men and women who are classified by their BMI as overweight/obese, and among women classified as “normal,” so that greater concern led to a stronger relationship between BMI and depressive effects. Women are much more vulnerable to “fat” concerns overall, and this accordingly heightens their depressive risk relative to men as they go up in weight across both normal and overweight/obese weight categories.

Moreover, while those with the lowest BMI have the highest depressive levels, our analysis shows that weight concerns are apparently not the reason why. That is, concerns about being thin (마른) did mediate the relationship between underweight and depression in either gender.

This brings us to the question of what the South Korean men's pattern means, compared to that of women. Specifically, are we seeing the start of a trend whereby men are becoming similarly susceptible to body concerns as a driver of depression? The broader context seems to suggest that. As noted, deep concern with appearance is an everyday part of life in East Asia, perhaps especially so in Korea (Jackson, Keel, & Lee, 2006; Pike et al., 2014). Altering the body through dieting and cosmetic surgery, once thought to be the practice of women, is increasingly undertaken by men (Holliday and Elfving‐Hwang, 2012). Indeed, the expectation for men to be simultaneously tall, slim and muscular with ‘soft’ features (e.g., less angular jaw, no hair) is driving the increased consumption of body modifying products and services by men (Holliday and Elfving‐Hwang, 2012; Schwekendiek et al., 2013). This particular look for men has been popular in East Asia since the mid‐1990s (cf. Miller, 2006), and by 2009 the top male celebrities (aged 25–29 years) in South Korea had an average height of 180.2 cm and an average BMI of 20.2 (Schwekendiek et al., 2013). These ideal men's lives are directly contradicted by real men's lives as reported in newspapers. Company men are putting on weight at alarming rates (Lee, 2015). This is attributed to long hours sitting at a desk followed by substantial eating and drinking events in the evening.

Further, the non‐finding related to “thin” concerns for those who are underweight might also seem to be an obvious finding in this specific anti‐fat cultural context. Yet it still bears consideration as a general point as we work toward a broader set of theories of how weight stigmas might be indirectly shaping a globalized human biology, due to the almost complete lack of existing studies considering weight‐stigma at both ends of the human body distribution outside of the Anglosphere. Comparing the findings here to the recent analysis of large Guatemalan nationally‐representative samples suggest there is much work still to be done to begin building any basic theory for how the mechanisms underlying thin‐concern and fat‐concern (as failure to meet body norms) might differently link to mental health across diverse socio‐cultural contexts.

A further contribution to theory building related to understanding the biology of stigma and social exclusion is through demonstrating that weight stigma is exerting apparent negative effects on health and human biology outside of the Anglosphere. While the case of body concerns in South Korea—where cosmetic surgery is relatively normalized and weight concerns abound even while obesity is relatively rare—may be extreme at first glance, this clarifies the mechanism through which extreme social norms driving an imagined biology (“being too fat”) can be powerful in shaping actual biology on another dimension (depression/depressive symptoms).

More generally, better understanding the biocultural bases of depression remains a high global public health priority. As we see here in the Korean case, there are many often invisible or underappreciated factors that provide important mechanisms underpinning depressive risk in diverse cultural contexts. Here, weight norms are shown to be one that is influential. The most recent Global Burden of Disease study highlights the role of depressive disorders as the largest nonfatal contributor to the global burden of disease (Ferrari, Charlson, Norman, Patten, Freedman, Murray, … Whiteford, 2013); understanding why depressive risk is distributed in relation to social norms and arrangements is important to addressing it as a massive global public health concern. These findings also suggest that as overweight and obesity rates continue to rise in Korea (as they are predicted to), so should overall depressive risk for both genders (even if women are overall at greater risk). While the negative effects of weight‐stigma in creating health disparities is increasingly appreciated and addressed as a policy concern in the U.S. (e.g., Puhl and Heuer, 2010), this is not the case in South Korea. This analysis suggests similar efforts to raise awareness and address stigma may be warranted in South Korea as part of a broader public health strategy, and should be focused on men as well as women.

In terms of broader theory building related to the biocultural construction of depression, these findings suggest several important refinements. First, epidemiological studies have posited that body concern helps explain the female preponderance of depressive risk in industrialized societies. Here we show that there are cultural contexts in which men can be vulnerable to concerns of failing to meet body norms as well, and that these can have similar apparent impacts on (mental) health.

Of course, the effect of body concerns on depressive symptoms for men is subdued compared to those of women, being focused among men who are objectively (clinically) measured and categorized as overweight. As discussed above, women are significantly at greater risk overall in part because there remains gender‐differentiated norms and roles. Our analysis thus converges with a growing set of biocultural literature explaining how gender roles and expectations shape mental health (Hadley and Patil, 2006; Pike and Williams, 2006; Weaver, Worthman, DeCaro, & Madhu, 2015; Wutich, 2009; Wutich and Brewis, 2016; Wutich and Ragsdale, 2008), most specifically addressing the ways in which such gendered norms shape embodied risk (see also the Dressler, Oths, Balieiro, Ribeiro, & Dos Santos, 2012 study of BMI and cultural consonance in Brazil). This South Korean case provides a novel example of how failure to meet social norms related to body weight is apparently stressful, and how, when, and why that stress may or may not be gendered.

Studies like this, testing how globalized and localized over‐weight concerns and obesity‐related stigma shape mental health outside of the West, are also, of course, new and few (cf., Hackman et al., 2016). But these have the potential to provide a new lens for explicating how dynamic social norms become embodied, and more generally develop a dynamic, contemporary human biology that uses the whole body as a lens for understanding the complicated pathways of illness and suffering underlying local and global health disparities (Krieger, 2005; Worthman and Costello, 2009).

## Supporting information

Additional Supporting information may be found in the online version of this article.

Supporting information
