# Supplementary material for: Weight, gender, and depressive symptoms in South Korea
Source: Am J Hum Biol. 2017 Feb 5;29(4):e22972. doi: 10.1002/ajhb.22972 (PMC5573951; doi:10.1002/ajhb.22972)
Supplement: Supplementary file 1 — Supporting information [file AJHB-29-na-s001.docx]

| <Appendix 1a> Mediation Analysis Results, Female with Body Mass Index < 18.5, Korea, 2014 | | | | | | | | | |
| --- | --- | --- | --- | --- | --- | --- | --- | --- | --- |
|  | On PHQ-9 | | | On Weight concern | | | On PHQ-9 | | |
|  | B |  | S.E. | B |  | S.E. | B |  | S.E. |
| Body mass index (BMI) | -0.27 |  | 0.52 | -0.21 | *** | 0.06 | -0.14 |  | 0.53 |
| Weight concerns |  |  |  |  |  |  | 0.64 |  | 0.56 |
| Health-related factors |  |  |  |  |  |  |  |  |  |
| Self-assessed health | -1.72 | ** | 0.55 | 0.15 |  | 0.08 | -1.82 | ** | 0.55 |
| Stress | 3.46 | *** | 0.74 | 0.13 |  | 0.11 | 3.38 | *** | 0.76 |
| Chronic disease | 0.02 |  | 2.21 | 0.26 |  | 0.21 | -0.14 |  | 2.26 |
| *Life quality measures* |  |  |  |  |  |  |  |  |  |
| Physical activity score | -0.59 |  | 1.61 | 0.05 |  | 0.19 | -0.62 |  | 1.62 |
| Self-management score | 5.17 |  | 3.23 | -0.45 | * | 0.23 | 5.45 |  | 3.32 |
| Normal activity score | -5.48 | * | 2.55 | -0.45 | * | 0.22 | -5.19 | * | 2.59 |
| Pain or discomfort score | -1.60 |  | 1.27 | -0.27 | * | 0.13 | -1.42 |  | 1.28 |
| Socioeconomic factors |  |  |  |  |  |  |  |  |  |
| Household income (10,000 won) | -0.10 |  | 0.42 | 0.07 |  | 0.06 | -0.14 |  | 0.41 |
| Own a house or an apartment | -0.59 |  | 0.72 | 0.06 |  | 0.09 | -0.63 |  | 0.72 |
| *Occupation status ^a^* |  |  |  |  |  |  |  |  |  |
| Non-regular | -1.26 |  | 0.86 | 0.14 |  | 0.13 | -1.35 |  | 0.87 |
| Self-employed or else | -0.29 |  | 1.36 | 0.21 |  | 0.16 | -0.42 |  | 1.37 |
| Not working or unemployed | 0.19 |  | 1.03 | 0.18 |  | 0.14 | 0.07 |  | 1.01 |
| *Respondent’s education ^b^* |  |  |  |  |  |  |  |  |  |
| Less than high school | 1.25 |  | 1.67 | -0.17 |  | 0.20 | 1.36 |  | 1.68 |
| College or above | -0.85 |  | 0.64 | 0.04 |  | 0.10 | -0.88 |  | 0.64 |
| *Parents’ education* |  |  |  |  |  |  |  |  |  |
| College or above, father | 0.60 |  | 0.87 | 0.02 |  | 0.12 | 0.59 |  | 0.88 |
| College or above, mother | -0.99 |  | 1.05 | -0.04 |  | 0.15 | -0.96 |  | 1.05 |
| Weight management ^c^ |  |  |  |  |  |  |  |  |  |
| Tried to gain | -0.35 |  | 0.96 | 0.32 | ** | 0.11 | -0.55 |  | 0.91 |
| Tried to lose | -0.21 |  | 0.87 | -0.31 | * | 0.13 | -0.02 |  | 0.92 |
| Demographic factors |  |  |  |  |  |  |  |  |  |
| *Marital status ^d^* |  |  |  |  |  |  |  |  |  |
| Never married | 0.20 |  | 1.30 | 0.06 |  | 0.14 | 0.16 |  | 1.32 |
| Divorced, widowed, or separated | 3.37 |  | 1.48 | 0.44 | * | 0.18 | 3.09 | * | 1.54 |
| *Age group ^e^* |  |  |  |  |  |  |  |  |  |
| Between 30 and 39 | 0.82 |  | 1.27 | 0.19 |  | 0.14 | 0.70 |  | 1.26 |
| Between 40 and 49 | -0.37 |  | 1.49 | 0.24 |  | 0.17 | -0.52 |  | 1.51 |
| Between 50 and 59 | -2.61 |  | 1.70 | 0.30 |  | 0.19 | -2.80 |  | 1.74 |
| Over 60 | -0.72 |  | 3.37 | 0.38 |  | 0.27 | -0.96 |  | 3.32 |
| Intercept | 12.63 |  | 8.30 | 0.95 |  | 0.71 | 12.03 |  | 8.55 |
| R^2^ | .58 | | | .47 | | | .58 | | |
| N *^f^* | 173 | | | 173 | | | 173 | | |
| Note: * *p* <.05; ** *p* <.01; *** *p* <.001, two tailed; adjusted for sample weight; Reference categories: *^a^ tried to keep weight or do nothing* as a reference group; *^b^ regular work* as a reference group; ^c^ *high school graduate* as a reference group; ^d^ *currently married* as a reference group; ^e^ *between 19 and 29 years old* as a reference group; ^f^ unweighted sample size. | | | | | | | | | |

| <Appendix 1b> Mediation Analysis Results, Male with Body Mass Index < 18.5, Korea, 2014 | | | | | | | | | |
| --- | --- | --- | --- | --- | --- | --- | --- | --- | --- |
|  | On PHQ-9 | | | On Weight concern | | | On PHQ-9 | | |
|  | B |  | S.E. | B |  | S.E. | B |  | S.E. |
| Body mass index (BMI) | -0.38 |  | 0.70 | -0.06 |  | 0.08 | -0.35 |  | 0.73 |
| Weight concerns |  |  |  |  |  |  | 0.59 |  | 1.59 |
| Health-related factors |  |  |  |  |  |  |  |  |  |
| Self-assessed health | -2.29 | ** | 0.71 | -0.13 |  | 0.08 | -2.21 | ** | 0.83 |
| Stress | 2.23 |  | 1.24 | 0.16 |  | 0.18 | 2.14 |  | 1.08 |
| Chronic disease | -0.68 |  | 1.48 | -0.80 | ** | 0.25 | -0.21 |  | 1.94 |
| *Life quality measures* |  |  |  |  |  |  |  |  |  |
| Physical activity score | -0.14 |  | 2.20 | -0.48 |  | 0.29 | 0.14 |  | 2.52 |
| Self-management score | -6.44 | * | 3.11 | -0.04 |  | 0.36 | -6.42 | * | 3.10 |
| Normal activity score | 0.33 |  | 2.76 | 0.17 |  | 0.19 | 0.23 |  | 2.78 |
| Pain or discomfort score | -0.71 |  | 2.25 | 0.09 |  | 0.22 | -0.76 |  | 2.26 |
| Socioeconomic factors |  |  |  |  |  |  |  |  |  |
| Household income (10,000 won) | -0.81 |  | 0.53 | 0.10 |  | 0.07 | -0.87 |  | 0.58 |
| Own a house or an apartment | 0.82 |  | 1.06 | 0.14 |  | 0.13 | 0.74 |  | 1.22 |
| *Occupation status ^a^* |  |  |  |  |  |  |  |  |  |
| Non-regular | 0.91 |  | 1.49 | 0.11 |  | 0.24 | 0.85 |  | 1.58 |
| Self-employed or else | 1.84 |  | 1.60 | -0.25 |  | 0.17 | 1.99 |  | 1.57 |
| Not working or unemployed | -0.28 |  | 1.17 | 0.21 |  | 0.16 | -0.40 |  | 1.22 |
| *Respondent’s education ^b^* |  |  |  |  |  |  |  |  |  |
| Less than high school | -1.37 |  | 1.64 | -0.63 | *** | 0.14 | -1.00 |  | 1.81 |
| College or above | 0.14 |  | 1.77 | -0.46 |  | 0.23 | 0.41 |  | 1.77 |
| *Parents’ education* |  |  |  |  |  |  |  |  |  |
| College or above, father | 4.54 | ** | 1.35 | -0.61 | ** | 0.22 | 4.90 | ** | 1.64 |
| College or above, mother | -3.20 | * | 1.55 | 0.11 |  | 0.26 | -3.26 | * | 1.44 |
| Weight management ^c^ |  |  |  |  |  |  |  |  |  |
| Tried to gain | 1.39 |  | 1.01 | -0.11 |  | 0.19 | 1.45 |  | 1.02 |
| Tried to lose | -1.37 |  | 3.74 | -0.19 |  | 0.40 | -1.26 |  | 3.77 |
| Demographic factors |  |  |  |  |  |  |  |  |  |
| *Marital status ^d^* |  |  |  |  |  |  |  |  |  |
| Never married | 2.55 |  | 2.27 | -0.20 |  | 0.17 | 2.66 |  | 2.22 |
| Divorced, widowed, or separated | 2.24 |  | 2.12 | 0.06 |  | 0.24 | 2.20 |  | 2.15 |
| *Age group ^e^* |  |  |  |  |  |  |  |  |  |
| Between 30 and 39 | 2.03 |  | 1.05 | 0.54 | ** | 0.17 | 1.71 |  | 0.90 |
| Between 40 and 49 | 2.39 |  | 1.46 | 0.84 | *** | 0.17 | 1.90 |  | 1.86 |
| Between 50 and 59 | -0.14 |  | 1.79 | 0.45 |  | 0.35 | -0.41 |  | 1.52 |
| Over 60 | 2.67 |  | 1.93 | 0.18 |  | 0.21 | 2.56 |  | 1.87 |
| Intercept | 22.41 | *** | 5.77 | 0.51 |  | 1.08 | 22.11 | *** | 5.78 |
| R^2^ | .60 | | | .62 | | | .61 | | |
| N *^f^* | 75 | | | 75 | | | 75 | | |
| Note: * *p* <.05; ** *p* <.01; *** *p* <.001, two tailed; adjusted for sample weight; Reference categories: *^a^ tried to keep weight or do nothing* as a reference group; *^b^ regular work* as a reference group; ^c^ *high school graduate* as a reference group; ^d^ *currently married* as a reference group; ^e^ *between 19 and 29 years old* as a reference group; ^f^ unweighted sample size. | | | | | | | | | |

| <Appendix 2a> Mediation Analysis Results, Female with Body Mass Index between 18.5 and 25, Korea, 2014 | | | | | | | | | |
| --- | --- | --- | --- | --- | --- | --- | --- | --- | --- |
|  | On PHQ-9 | | | On Weight concern | | | On PHQ-9 | | |
|  | B |  | S.E. | B |  | S.E. | B |  | S.E. |
| Body mass index (BMI) | -0.08 |  | 0.05 | 0.17 | *** | 0.01 | -0.17 | ** | 0.06 |
| Weight concerns |  |  |  |  |  |  | 0.54 | ** | 0.20 |
| Health-related factors |  |  |  |  |  |  |  |  |  |
| Self-assessed health | -0.88 | *** | 0.12 | -0.09 | *** | 0.02 | -0.84 | *** | 0.12 |
| Stress | 3.36 | *** | 0.21 | 0.05 |  | 0.03 | 3.33 | *** | 0.21 |
| Chronic disease | 0.13 |  | 0.25 | 0.00 |  | 0.04 | 0.13 |  | 0.25 |
| *Life quality measures* |  |  |  |  |  |  |  |  |  |
| Physical activity score | -0.93 | * | 0.46 | -0.02 |  | 0.05 | -0.93 | * | 0.45 |
| Self-management score | -0.78 |  | 0.62 | 0.11 |  | 0.06 | -0.82 |  | 0.61 |
| Normal activity score | -1.23 | * | 0.48 | 0.02 |  | 0.07 | -1.24 | ** | 0.48 |
| Pain or discomfort score | -0.81 | *** | 0.22 | 0.00 |  | 0.03 | -0.80 | *** | 0.22 |
| Socioeconomic factors |  |  |  |  |  |  |  |  |  |
| Household income (10,000 won) | -0.07 |  | 0.09 | 0.03 |  | 0.01 | -0.08 |  | 0.09 |
| Own a house or an apartment | -0.26 |  | 0.20 | -0.01 |  | 0.03 | -0.26 |  | 0.20 |
| *Occupation status ^a^* |  |  |  |  |  |  |  |  |  |
| Non-regular | 0.13 |  | 0.28 | -0.02 |  | 0.04 | 0.14 |  | 0.29 |
| Self-employed or else | 0.29 |  | 0.31 | 0.00 |  | 0.04 | 0.29 |  | 0.31 |
| Not working or unemployed | 0.39 |  | 0.25 | 0.01 |  | 0.03 | 0.39 |  | 0.26 |
| *Respondent’s education ^b^* |  |  |  |  |  |  |  |  |  |
| Less than high school | 0.12 |  | 0.28 | -0.01 |  | 0.04 | 0.12 |  | 0.29 |
| College or above | -0.16 |  | 0.20 | 0.01 |  | 0.03 | -0.16 |  | 0.20 |
| *Parents’ education* |  |  |  |  |  |  |  |  |  |
| College or above, father | 0.13 |  | 0.24 | 0.01 |  | 0.04 | 0.12 |  | 0.24 |
| College or above, mother | -0.52 |  | 0.32 | -0.01 |  | 0.05 | -0.51 |  | 0.33 |
| Weight management ^c^ |  |  |  |  |  |  |  |  |  |
| Tried to gain | 0.46 |  | 0.40 | 0.05 |  | 0.04 | 0.44 |  | 0.39 |
| Tried to lose | 0.18 |  | 0.19 | 0.13 | *** | 0.03 | 0.11 |  | 0.19 |
| Demographic factors |  |  |  |  |  |  |  |  |  |
| *Marital status ^d^* |  |  |  |  |  |  |  |  |  |
| Never married | 0.27 |  | 0.42 | 0.14 | ** | 0.05 | 0.19 |  | 0.42 |
| Divorced, widowed, or separated | -0.10 |  | 0.27 | 0.05 |  | 0.04 | -0.13 |  | 0.27 |
| *Age group ^e^* |  |  |  |  |  |  |  |  |  |
| Between 30 and 39 | -0.34 |  | 0.42 | 0.05 |  | 0.05 | -0.37 |  | 0.42 |
| Between 40 and 49 | -0.59 |  | 0.47 | -0.03 |  | 0.06 | -0.58 |  | 0.48 |
| Between 50 and 59 | -0.50 |  | 0.48 | -0.11 |  | 0.06 | -0.43 |  | 0.48 |
| Over 60 | -0.92 |  | 0.57 | -0.27 | *** | 0.06 | -0.77 |  | 0.58 |
| Intercept | 11.93 | *** | 1.47 | -0.14 |  | 0.16 | 11.98 | *** | 1.46 |
| R^2^ | .33 | | | .37 | | | .33 | | |
| N *^f^* | 2,179 | | | 2,179 | | | 2,179 | | |
| Note: * *p* <.05; ** *p* <.01; *** *p* <.001, two tailed; adjusted for sample weight; Reference categories: *^a^ tried to keep weight or do nothing* as a reference group; *^b^ regular work* as a reference group; ^c^ *high school graduate* as a reference group; ^d^ *currently married* as a reference group; ^e^ *between 19 and 29 years old* as a reference group; ^f^ unweighted sample size. | | | | | | | | | |

| <Appendix 2b> Mediation Analysis Results, Male with Body Mass Index between 18.5 and 25, Korea, 2014 | | | | | | | | | |
| --- | --- | --- | --- | --- | --- | --- | --- | --- | --- |
|  | On PHQ-9 | | | On Weight concern | | | On PHQ-9 | | |
|  | B |  | S.E. | B |  | S.E. | B |  | S.E. |
| Body mass index (BMI) | 0.07 |  | 0.06 | 0.07 | *** | 0.01 | 0.10 |  | 0.06 |
| Weight concerns |  |  |  |  |  |  | -0.40 |  | 0.22 |
| Health-related factors |  |  |  |  |  |  |  |  |  |
| Self-assessed health | -0.80 | *** | 0.11 | -0.03 |  | 0.01 | -0.81 | *** | 0.10 |
| Stress | 2.45 | *** | 0.31 | 0.02 |  | 0.03 | 2.46 | *** | 0.30 |
| Chronic disease | 0.07 |  | 0.24 | -0.04 |  | 0.03 | 0.06 |  | 0.24 |
| *Life quality measures* |  |  |  |  |  |  |  |  |  |
| Physical activity score | -0.33 |  | 0.50 | 0.02 |  | 0.05 | -0.32 |  | 0.50 |
| Self-management score | -0.06 |  | 0.99 | 0.11 |  | 0.07 | -0.01 |  | 0.99 |
| Normal activity score | -0.89 |  | 0.74 | -0.03 |  | 0.08 | -0.91 |  | 0.74 |
| Pain or discomfort score | -1.31 | *** | 0.27 | 0.01 |  | 0.03 | -1.31 | *** | 0.27 |
| Socioeconomic factors |  |  |  |  |  |  |  |  |  |
| Household income (10,000 won) | -0.03 |  | 0.13 | -0.01 |  | 0.02 | -0.04 |  | 0.13 |
| Own a house or an apartment | -0.43 | * | 0.20 | 0.02 |  | 0.03 | -0.42 | * | 0.20 |
| *Occupation status ^a^* |  |  |  |  |  |  |  |  |  |
| Non-regular | 0.32 |  | 0.28 | 0.07 |  | 0.03 | 0.34 |  | 0.27 |
| Self-employed or else | -0.03 |  | 0.22 | 0.03 |  | 0.03 | -0.01 |  | 0.22 |
| Not working or unemployed | 0.35 |  | 0.34 | 0.08 |  | 0.04 | 0.38 |  | 0.33 |
| *Respondent’s education ^b^* |  |  |  |  |  |  |  |  |  |
| Less than high school | 0.27 |  | 0.25 | 0.00 |  | 0.03 | 0.27 |  | 0.25 |
| College or above | -0.26 |  | 0.21 | 0.01 |  | 0.03 | -0.25 |  | 0.20 |
| *Parents’ education* |  |  |  |  |  |  |  |  |  |
| College or above, father | -0.02 |  | 0.35 | -0.04 |  | 0.04 | -0.04 |  | 0.35 |
| College or above, mother | -0.15 |  | 0.47 | 0.14 | * | 0.06 | -0.09 |  | 0.47 |
| Weight management ^c^ |  |  |  |  |  |  |  |  |  |
| Tried to gain | 0.34 |  | 0.32 | 0.02 |  | 0.02 | 0.34 |  | 0.32 |
| Tried to lose | 0.06 |  | 0.23 | 0.15 | *** | 0.04 | 0.12 |  | 0.23 |
| Demographic factors |  |  |  |  |  |  |  |  |  |
| *Marital status ^d^* |  |  |  |  |  |  |  |  |  |
| Never married | 0.14 |  | 0.37 | -0.05 |  | 0.04 | 0.12 |  | 0.37 |
| Divorced, widowed, or separated | -0.27 |  | 0.45 | -0.04 |  | 0.05 | -0.29 |  | 0.45 |
| *Age group ^e^* |  |  |  |  |  |  |  |  |  |
| Between 30 and 39 | -0.10 |  | 0.46 | -0.01 |  | 0.04 | -0.10 |  | 0.45 |
| Between 40 and 49 | -0.49 |  | 0.46 | 0.01 |  | 0.04 | -0.49 |  | 0.46 |
| Between 50 and 59 | -0.55 |  | 0.46 | -0.06 |  | 0.05 | -0.58 |  | 0.45 |
| Over 60 | -1.00 | * | 0.48 | -0.06 |  | 0.05 | -1.02 | * | 0.47 |
| Intercept | 9.13 | *** | 2.43 | -0.15 |  | 0.14 | 9.07 | *** | 2.42 |
| R^2^ | .30 | | | .20 | | | .30 | | |
| N *^f^* | 1,430 | | | 1,430 | | | 1,430 | | |
| Note: * *p* <.05; ** *p* <.01; *** *p* <.001, two tailed; adjusted for sample weight; Reference categories: *^a^ tried to keep weight or do nothing* as a reference group; *^b^ regular work* as a reference group; ^c^ *high school graduate* as a reference group; ^d^ *currently married* as a reference group; ^e^ *between 19 and 29 years old* as a reference group; ^f^ unweighted sample size. | | | | | | | | | |

| <Appendix 3a> Mediation Analysis Results, Female with Body Mass Index ≥ 25, Korea, 2014 | | | | | | | | | |
| --- | --- | --- | --- | --- | --- | --- | --- | --- | --- |
|  | On PHQ-9 | | | On Weight concern | | | On PHQ-9 | | |
|  | B |  | S.E. | B |  | S.E. | B |  | S.E. |
| Body mass index (BMI) | 0.19 | ** | 0.06 | 0.09 | *** | 0.01 | 0.12 |  | 0.07 |
| Weight concerns |  |  |  |  |  |  | 0.75 | ** | 0.26 |
| Health-related factors |  |  |  |  |  |  |  |  |  |
| Self-assessed health | -0.72 | *** | 0.17 | -0.05 | * | 0.03 | -0.68 | *** | 0.17 |
| Stress | 3.47 | *** | 0.43 | 0.03 |  | 0.06 | 3.46 | *** | 0.41 |
| Chronic disease | -0.24 |  | 0.35 | 0.14 | * | 0.06 | -0.34 |  | 0.35 |
| *Life quality measures* |  |  |  |  |  |  |  |  |  |
| Physical activity score | 0.05 |  | 0.44 | 0.06 |  | 0.08 | 0.01 |  | 0.42 |
| Self-management score | 0.41 |  | 0.97 | 0.13 |  | 0.13 | 0.31 |  | 0.94 |
| Normal activity score | -1.91 | * | 0.75 | -0.18 | * | 0.09 | -1.77 | * | 0.72 |
| Pain or discomfort score | -1.73 | *** | 0.31 | -0.07 |  | 0.05 | -1.68 | *** | 0.30 |
| Socioeconomic factors |  |  |  |  |  |  |  |  |  |
| Household income (10,000 won) | -0.52 | ** | 0.19 | 0.02 |  | 0.03 | -0.53 | ** | 0.19 |
| Own a house or an apartment | 0.12 |  | 0.30 | -0.08 |  | 0.05 | 0.18 |  | 0.30 |
| *Occupation status ^a^* |  |  |  |  |  |  |  |  |  |
| Non-regular | -0.20 |  | 0.44 | -0.01 |  | 0.07 | -0.19 |  | 0.44 |
| Self-employed or else | 0.49 |  | 0.52 | -0.04 |  | 0.09 | 0.52 |  | 0.51 |
| Not working or unemployed | 0.93 |  | 0.47 | 0.01 |  | 0.07 | 0.92 |  | 0.48 |
| *Respondent’s education ^b^* |  |  |  |  |  |  |  |  |  |
| Less than high school | -0.22 |  | 0.37 | -0.10 |  | 0.06 | -0.15 |  | 0.37 |
| College or above | 0.39 |  | 0.37 | -0.06 |  | 0.06 | 0.43 |  | 0.37 |
| *Parents’ education* |  |  |  |  |  |  |  |  |  |
| College or above, father | -0.29 |  | 0.55 | -0.01 |  | 0.09 | -0.29 |  | 0.56 |
| College or above, mother | 0.34 |  | 0.87 | 0.04 |  | 0.15 | 0.31 |  | 0.88 |
| Weight management ^c^ |  |  |  |  |  |  |  |  |  |
| Tried to gain | 2.81 |  | 2.28 | 0.14 |  | 0.23 | 2.71 |  | 2.27 |
| Tried to lose | -0.14 |  | 0.29 | 0.13 | * | 0.05 | -0.23 |  | 0.29 |
| Demographic factors |  |  |  |  |  |  |  |  |  |
| *Marital status ^d^* |  |  |  |  |  |  |  |  |  |
| Never married | -0.92 |  | 0.83 | -0.15 |  | 0.10 | -0.81 |  | 0.83 |
| Divorced, widowed, or separated | 1.35 | * | 0.53 | -0.02 |  | 0.07 | 1.36 | * | 0.53 |
| *Age group ^e^* |  |  |  |  |  |  |  |  |  |
| Between 30 and 39 | -1.93 | ** | 0.71 | -0.09 |  | 0.11 | -1.86 | * | 0.74 |
| Between 40 and 49 | -1.95 | * | 0.87 | -0.15 |  | 0.10 | -1.84 | * | 0.89 |
| Between 50 and 59 | -2.22 | ** | 0.84 | -0.29 | * | 0.11 | -2.00 | * | 0.86 |
| Over 60 | -2.97 | ** | 0.89 | -0.52 | *** | 0.12 | -2.58 | ** | 0.93 |
| Intercept | 13.88 | *** | 2.03 | 0.47 |  | 0.28 | 13.52 | *** | 1.96 |
| R^2^ | .44 | | | .33 | | | .45 | | |
| N *^f^* | 900 | | | 900 | | | 900 | | |
| Note: * *p* <.05; ** *p* <.01; *** *p* <.001, two tailed; adjusted for sample weight; Reference categories: *^a^ tried to keep weight or do nothing* as a reference group; *^b^ regular work* as a reference group; ^c^ *high school graduate* as a reference group; ^d^ *currently married* as a reference group; ^e^ *between 19 and 29 years old* as a reference group; ^f^ unweighted sample size. | | | | | | | | | |

| <Appendix 3b> Mediation Analysis Results, Male with Body Mass Index ≥ 25, Korea, 2014 | | | | | | | | | |
| --- | --- | --- | --- | --- | --- | --- | --- | --- | --- |
|  | On PHQ-9 | | | On Weight concern | | | On PHQ-9 | | |
|  | B |  | S.E. | B |  | S.E. | B |  | S.E. |
| Body mass index (BMI) | 0.01 |  | 0.05 | 0.12 | *** | 0.01 | -0.06 |  | 0.06 |
| Weight concerns |  |  |  |  |  |  | 0.62 | ** | 0.23 |
| Health-related factors |  |  |  |  |  |  |  |  |  |
| Self-assessed health | -0.71 | *** | 0.15 | -0.05 |  | 0.03 | -0.68 | *** | 0.15 |
| Stress | 1.99 | *** | 0.34 | -0.04 |  | 0.05 | 2.02 | *** | 0.33 |
| Chronic disease | 0.17 |  | 0.31 | 0.07 |  | 0.04 | 0.13 |  | 0.31 |
| *Life quality measures* |  |  |  |  |  |  |  |  |  |
| Physical activity score | 1.43 | * | 0.64 | -0.06 |  | 0.09 | 1.47 | * | 0.65 |
| Self-management score | -0.36 |  | 0.74 | 0.22 |  | 0.16 | -0.50 |  | 0.75 |
| Normal activity score | -2.45 | ** | 0.91 | -0.02 |  | 0.13 | -2.44 | ** | 0.90 |
| Pain or discomfort score | -1.26 | ** | 0.46 | 0.01 |  | 0.05 | -1.27 | ** | 0.46 |
| Socioeconomic factors |  |  |  |  |  |  |  |  |  |
| Household income (10,000 won) | -0.09 |  | 0.20 | 0.02 |  | 0.02 | -0.10 |  | 0.21 |
| Own a house or an apartment | -0.59 | * | 0.28 | 0.04 |  | 0.05 | -0.62 | * | 0.28 |
| *Occupation status ^a^* |  |  |  |  |  |  |  |  |  |
| Non-regular | 0.21 |  | 0.32 | -0.03 |  | 0.07 | 0.23 |  | 0.32 |
| Self-employed or else | 0.06 |  | 0.26 | -0.08 |  | 0.06 | 0.11 |  | 0.26 |
| Not working or unemployed | 0.34 |  | 0.42 | -0.13 | * | 0.07 | 0.41 |  | 0.42 |
| *Respondent’s education ^b^* |  |  |  |  |  |  |  |  |  |
| Less than high school | -0.49 |  | 0.41 | -0.08 |  | 0.06 | -0.45 |  | 0.40 |
| College or above | -0.19 |  | 0.26 | 0.03 |  | 0.05 | -0.21 |  | 0.26 |
| *Parents’ education* |  |  |  |  |  |  |  |  |  |
| College or above, father | -0.17 |  | 0.36 | -0.04 |  | 0.09 | -0.15 |  | 0.35 |
| College or above, mother | -0.03 |  | 0.53 | 0.16 |  | 0.12 | -0.13 |  | 0.55 |
| Weight management ^c^ |  |  |  |  |  |  |  |  |  |
| Tried to gain | 2.85 |  | 2.59 | -0.28 |  | 0.23 | 3.03 |  | 2.49 |
| Tried to lose | -0.18 |  | 0.25 | 0.07 |  | 0.04 | -0.23 |  | 0.25 |
| Demographic factors |  |  |  |  |  |  |  |  |  |
| *Marital status ^d^* |  |  |  |  |  |  |  |  |  |
| Never married | 0.82 |  | 0.43 | 0.20 | * | 0.08 | 0.70 |  | 0.42 |
| Divorced, widowed, or separated | 0.75 |  | 0.68 | 0.04 |  | 0.09 | 0.73 |  | 0.65 |
| *Age group ^e^* |  |  |  |  |  |  |  |  |  |
| Between 30 and 39 | 0.51 |  | 0.54 | -0.09 |  | 0.10 | 0.57 |  | 0.53 |
| Between 40 and 49 | 0.00 |  | 0.56 | -0.14 |  | 0.11 | 0.09 |  | 0.56 |
| Between 50 and 59 | 0.32 |  | 0.61 | -0.19 |  | 0.12 | 0.43 |  | 0.61 |
| Over 60 | 0.13 |  | 0.66 | -0.21 |  | 0.12 | 0.26 |  | 0.66 |
| Intercept | 8.83 | *** | 1.92 | -0.21 |  | 0.27 | 8.95 | *** | 1.94 |
| R^2^ | .31 | | | .34 | | | .32 | | |
| N *^f^* | 875 | | | 875 | | | 875 | | |
| Note: * *p* <.05; ** *p* <.01; *** *p* <.001, two tailed; adjusted for sample weight; Reference categories: *^a^ tried to keep weight or do nothing* as a reference group; *^b^ regular work* as a reference group; ^c^ *high school graduate* as a reference group; ^d^ *currently married* as a reference group; ^e^ *between 19 and 29 years old* as a reference group; ^f^ unweighted sample size. | | | | | | | | | |
